# Peeking behind the carbocation: identification of (alternative) catalytic bases in the class II active site of conifer resin acid diterpene synthases

**DOI:** 10.1042/BCJ20250232

**Published:** 2026-04-28

**Authors:** Ahmed M.A.A. Raslan, Reuben J. Peters

**Affiliations:** Roy J. Carver Department of Biochemistry, Biophysics & Molecular Biology, Iowa State University, Ames, IA 50011, U.S.A.

**Keywords:** diterpene cyclase, enzymatic structure-function, resin acid

## Abstract

Class II diterpene cyclases (DTCs) initiate biosynthesis of the labdane-related diterpenoids (LRDs), utilizing an acid-base mechanism to catalyze bicyclization of the general diterpenoid precursor (*E,E,E*)-geranylgeranyl pyrophosphate (**1**), most often producing the eponymous labdadienyl/copalyl pyrophosphate (CPP, **2**). Prominent among the LRDs and terpenoids more generally are the conifer resin acids. The abietaenol synthase from *Abies grandis* (*Ag*AS), due in part to crystallographic structural analysis, serves as a model for the DTCs initiating resin acid biosynthesis, with such activity having been conserved for over 300 million years. Previous work suggests that a hydrogen-bonded tyrosine-histidine pair in its DTC active site serves as the catalytic base, in part because the substitution of aspartate for the histidine or phenylalanine for the tyrosine leads to the incorporation/addition of water and the production of labda-13-en-8α-ol-15-yl pyrophosphate (LPP, **3**). However, the exact identity of the catalytic base in the native reaction, as well as any alternative base(s) enabling the production of **3** and 7-endo-CPP (**4**) in the histidine to aspartate mutant, remains unknown. Here, the *TerDockin* computational approach, combining quantum chemical modeling with computational docking, was applied to the *Ag*AS DTC active site. This not only indicated the Tyr hydroxyl group serves as the native catalytic base but also surprisingly found a serine capable of serving as an alternative base for the production of **3** and a tyrosine serving as the alternative base for the production of **4**, as supported by mutational analysis in *Ag*AS. This provides mechanistic insight and further validates the *TerDockin* approach to investigation of these important enzymes.

## Introduction

Labdane-related diterpenoids (LRDs) are defined by the cyclization reaction catalyzed by class II diterpene cyclases (DTCs), specifically their common generation of an eponymous labdane-type carbocation intermediate [1]. These enzymes are then of central importance to the biosynthesis of this large superfamily, which contains over 15,000 known natural products [2]. While their prevalence, particularly in plants, can be attributed to the requisite role of the gibberellin phytohormones [3,4], it was conifer resin acids that were the first recognized members of this superfamily. Indeed, abietic acid served a central role in the elucidation of terpenoid chemistry more generally—e.g., the derivation of the isoprene rule [5].

Notably, the plant terpene synthase (TPS) family has been found to stem from the fusion of a DTC with a subsequently acting class I diterpene synthase (DTS) [6], with the first example of such a bifunctional DTC-DTS also derived from resin acid biosynthesis. Specifically, that from grand fir (*Abies grandis*), originally thought to produce abietadiene [7], but now verified to serve as an abietaenol synthase (*Ag*AS) [8], consistent with those from other conifers [9]. This was the founding member of the TPS-d subfamily [10], although it falls more specifically within the d3 clade that contains all the known DTC-DTSs involved in conifer resin acid biosynthesis along with others involved in the production of distinct LRDs [11]. Due to its early discovery, as well as x-ray crystallographic analysis of an apo structure [12], *Ag*AS has become the model for this TPS-d3 clade.

DTCs carry out bicyclization of the general diterpenoid precursor (E,E,E)-geranylgeranyl pyrophosphate (GGPP, **1**) via an acid-base mechanism, with the catalytic acid provided by the characteristic DxDD motif in which the ‘middle’ aspartic acid protonates the terminal olefin to initiate a carbocation cascade [13]. By contrast, the catalytic base varies as, despite generally producing labda-8(17),13*E*-dien-15-PP—commonly termed CPP (**2**), the observed variation in product outcome (both stereo- as well as regio-chemical) necessitates the use of differentially positioned functional groups for this purpose [14]. Thus, it has been difficult to identify the exact group serving this function in most DTCs, which can include hydroxyl groups (e.g., on amino acid side chains), especially given the geometric constraints on the relative positioning of carbocation to catalytic base [15].

Conifer resin acid biosynthesis proceeds through selective production of **2** by the DTC active site [9], which is then released and diffuses to the DTS active site as demonstrated with *Ag*AS [16]. The DTS active site, like all class I terpene synthases, contains a DDxxD motif required for binding the trio of divalent magnesium ion cofactors, with substitution of alanine for the first Asp sufficient to block such activity (e.g., D621A in *Ag*AS) [16]. Although the DTC and DTS active sites are distinct, contained in separate domains, the interface between them is critical for both [17].

**Figure 1:**
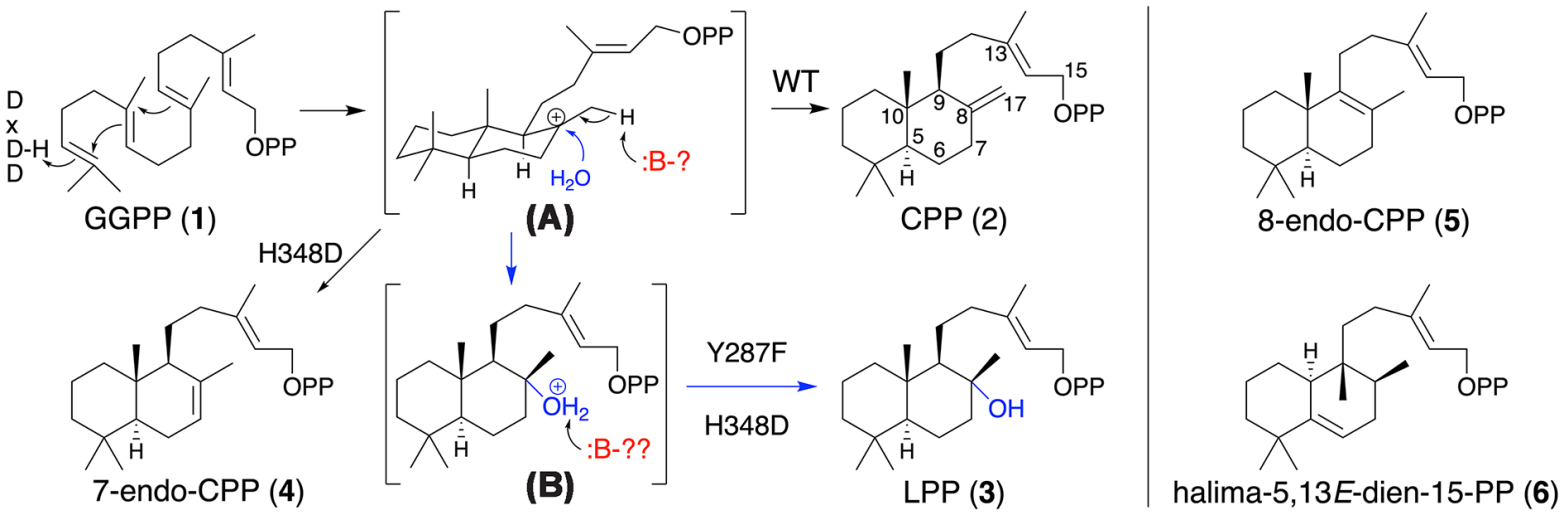
DTC reaction catalyzed by AgAS. DTC reaction catalyzed by *Ag*AS. Cyclization of GGPP (**1**) to carbocation intermediate (**A**) with wild-type (WT) produces CPP (**2**) utilizing an undefined catalytic base to deprotonate at C17, while both indicated mutants add water and deprotonate the resulting alkyloxonium (**B**) to produce LPP (**3**), and the H348D mutant also produces 7-endo-CPP (**4**), formed by deprotonation of **A** at C7. Also shown are the alternative products 8-endo-CPP (**5**), from deprotonation of **A** at C9, and halima-5,13*E*-dien-15-PP (**6**), formed from **A** via a series of 1,2-shifts (hydride from C9 to C8, methyl from C10 to C9, and then hydride from C5 to C10) with subsequent deprotonation at C6.

Following the determination of the *Ag*AS crystal structure [12], it was quickly realized that the DTC active site contained a histidine on the opposite side of the cleft from the DxDD motif that cooperatively serves as the catalytic acid. This was immediately hypothesized to serve as the catalytic base. Indeed, using the *Ag*AS:D621A (DTC-only) variant [16], it was found that substitution of this with alanine (H348A) led to predominant addition of water to the labda-13*E*-en-15-PP-8-yl carbocation intermediate (**A**), resulting in production of a hydroxylated derivative of CPP—i.e., labda-13-en-8α-ol-15-yl pyrophosphate (LPP) (**3**)—as well as small amounts of **2** and an olefin isomer determined to be labda-7,13*E*-dien-15-PP (7-endo-CPP, **4**)—i.e., by *Ag*AS:H348A/D621A (Figure 1) [14]. Moreover, based on earlier discovery of a native LPP synthase from *Abies balsamea* [18], which contained an aspartate at this position, it was found that such substitution led to more specific production of **3**, although **4** is still observed—i.e., by *Ag*AS:H348D/D621A [14].

In later work, examining the potential ability of the *Ag*AS DTS active site to act on **3** to produce the heterocyclic manoyl oxide, re-examination of the crystal structure revealed H348 is hydrogen-bonded to a tyrosine, substitution of phenylalanine for which led to more efficient production of manoyl oxide—i.e., by *Ag*AS:Y287F [19]. It is therefore hypothesized that this pair of residues cooperatively serves as the catalytic base in the production of **2**. However, which residue directly serves as the general base and if the other then serves to deprotonate the alkyloxonium (**B**) resulting from the incorporated water to produce **3** in the relevant variants remains unclear. Here, the *TerDockin* computational approach is applied to the *Ag*AS DTC active site to not only indicate Y287 serves as the native catalytic base but also a serine that seems to act as the catalytic base for the production of **3** in some cases, while a distinct tyrosine does so for the production of **4**.

## Results and discussion

To investigate the identity of the catalytic base in the *Ag*AS DTC active site, the *TerDockin* computational approach was applied. Specifically, a conformational library for intermediate **A** (see Supplementary Figure S1 for numbering), optimized using density function theory (mPW1PW91/6-31+g(d,p)), was docked into the active site using the Rosetta molecular modeling suite (see Supplementary Tables S1 and S2 for utilized constraints). Particularly given the lack of a well-defined water in the immediate vicinity of the hydrogen-bonded pair Y287 and H348, both of which have evidence suggesting activity as the catalytic base [14,19], the suitability of these residues to directly deprotonate **A** at C17 to produce CPP (**2**) was examined. To enable examination of H348 its optimal deprotonation angle was determined through transition state optimization using a theozyme system of imidazole and the methylcyclohexane with the predicted transition state further validated via intrinsic reaction coordinate (IRC) calculation (Supplementary Figure S2) (see the ‘Methods’ section for details on the minima, transition state, and IRC calculations). Examination of Y287 was guided by angle constraints from a previously optimized model system for tyrosine-mediated protonation in a (sesqui)terpene cyclization reaction [20]. For both residues, the distance from the proton-accepting atom to the carbon from which the proton will be abstracted was constrained to vary between 2.5 and 3.5 Å. All other constraints were the same (see Supplementary Tables S3 and S4 for utilized constraints).

**Figure 2:**
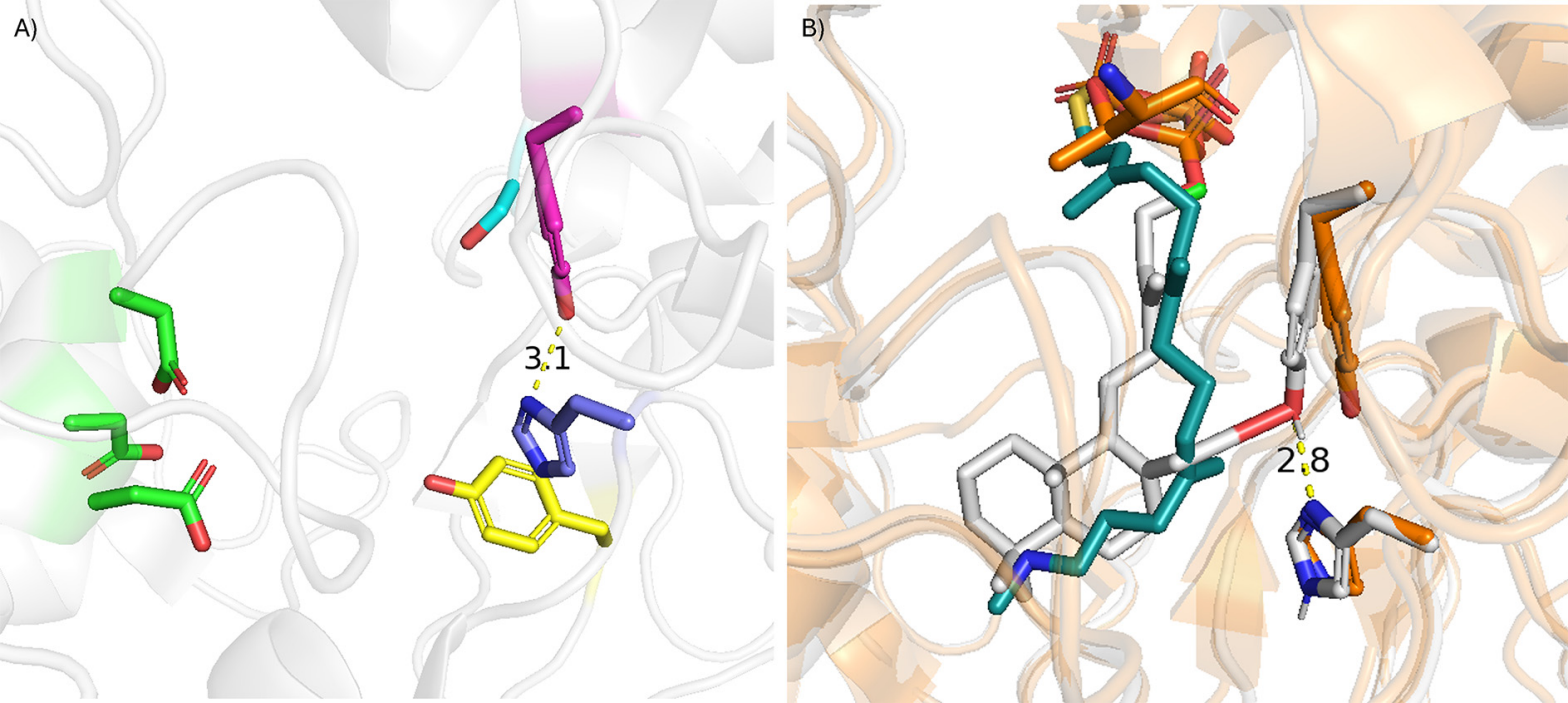
Catalytic bases in the AgAS DTC active site. Predicted catalytic bases in the *Ag*AS DTC active site. (**A**) *Ag*AS DTC active site, as defined by the DXDD motif, with residues investigated here shown. (**B**) Docking results for either Y287 or H348 acting as the catalytic base to deprotonate **A** and produce CPP (**2**) were combined and filtered based on constraint satisfaction, total protein energy (lowest 50%), and then interface energy (lowest 10%). A representative pose of the favored use of Y287 as the catalytic base (shown in grey) from the *Ag*AS apo structure (orange, PDB ID: 3S9V) overlaid with a substrate analog (shown in green) from the *Arabidopsis thaliana ent-*copalyl pyrophosphate synthase (*At*CPS) crystal structure (PDB ID: 4LIX), although it should be noted that the necessary magnesium co-factor is not present and nor is the analog in the pre-catalytic conformation for the *enantiomeric* CPP produced by *At*CPS.

The resulting docked poses were combined and filtered for conformance to the constraints (energy score <1), total energy score (lowest 50%), and protein-reactant interface energy score (lowest 10%). The results indicate that Y287 is the most likely candidate to act as the catalytic base during the deprotonation step, as >95% of the remaining poses were from use of this residue relative to those derived from use of H348 (Figure 2). This further implicates a mechanism in which H348 positions and activates Y287 through a hydrogen bond, analogous to the catalytic triad observed in serine proteases.

Similarly, the *TerDockin* approach was applied to investigate the mechanism of LPP (**3**) production in the Y287F and H348D variants. The relevant substitution was modeled for each, including docking a reactant water molecule as a separate ligand constrained to C8 of **A** to model this necessary addition (see Supplementary Table S5 for utilized constraints). The constraints representing the hydrogen bond involving the now missing H348 or Y287 were removed. All other constraints from the wild-type analysis were maintained. The poses that pass the filtering process then provide indications about the mechanism by which **3** is produced instead of **2**. Obviously, in the case of the Y287F variant, the usual catalytic base is no longer present. More interestingly, in the case of the H348D variant, the production of **2** is blocked by the reactant water, as it is predicted to be hydrogen-bonded to Y287, preventing its access to C17 of **A** (Supplementary Figure S3).

**Figure 3:**
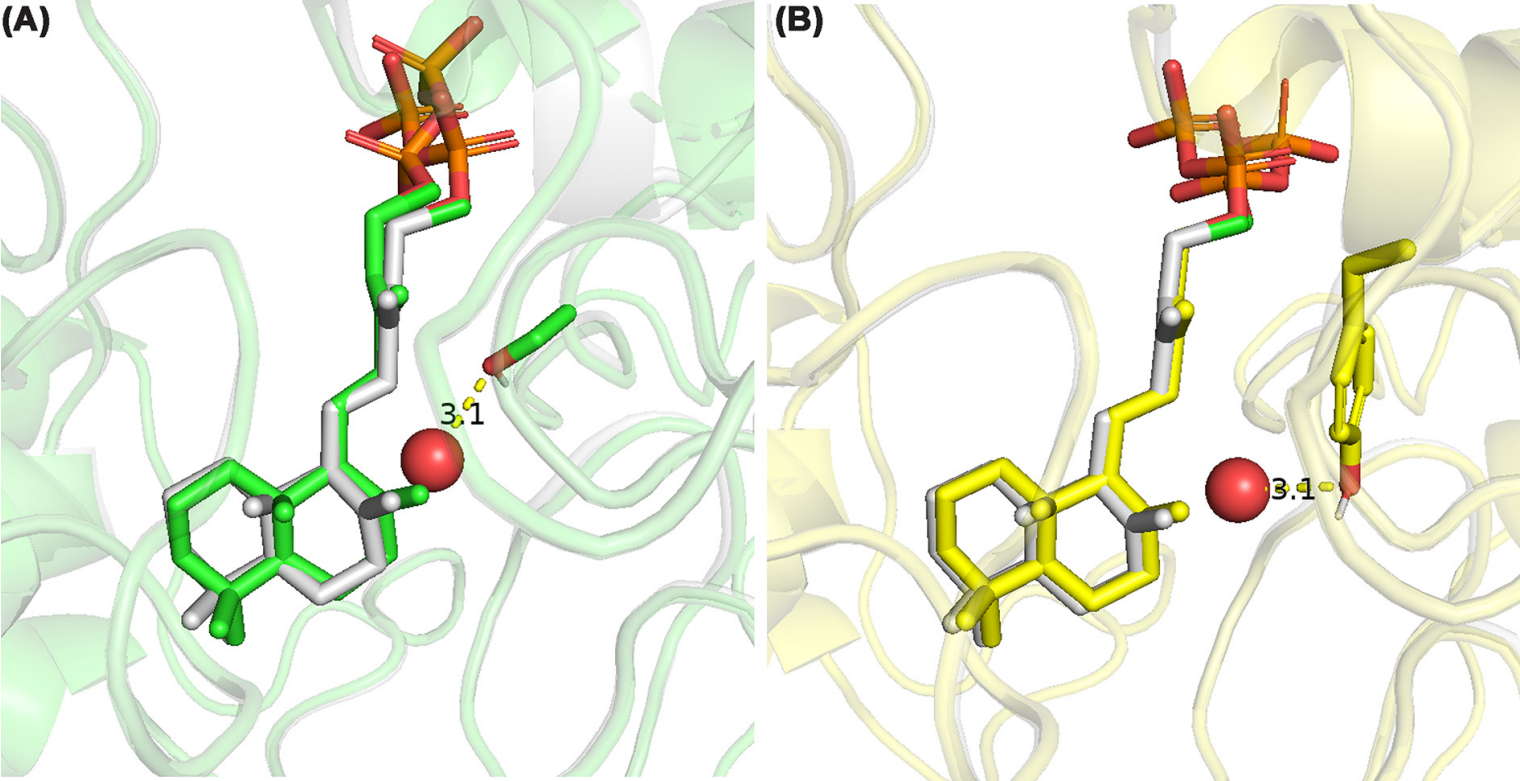
Catalytic bases for production of LPP. Representative docking poses show reactant water (red sphere) and intermediate **A** in *Ag*AS:H348D, illustrating positioning for addition and predicted hydrogen bond of the water to (**A**) S232 (green) or (**B**) Y287 (yellow), overlaid with wild type Y287 acting as a base to produce CPP (**2**) (grey), either of which may then act as the catalytic base in the production of LPP (**3**).

The *TerDockin* results were further examined to identify potential catalytic base(s) serving to deprotonate the alkyloxonium intermediate (**B**) resulting from the addition of water to **A** to yield **3**. In the H348D variant, analysis of the poses remaining after filtering (i.e., those most energetically favorable) revealed that the reactant water, although in 61% of the poses, is hydrogen-bonded to Y287, which may then serve as the catalytic base; only in 4% is D348 similarly appropriately positioned. In the remaining 35% of the poses, the water is proximal to a serine (S232), which may then serve as the catalytic base in such cases (Figure 3). Applying the same approach to the Y287F variant, we found that S232 is the most likely catalytic base for deprotonation of **B**, forming a hydrogen bond to the reactant water in 75% of the energetically favorable poses, compared with 25% for H348. Also of interest is the potential catalytic base for production of 7-endo-CPP (**4**)—i.e., deprotonation of **A** at C7—in the *Ag*AS:H348D mutant, which *TerDockin* suggested might be Y536 due to the proximity of its hydroxyl group (3.5 Å).

To experimentally investigate the significance of these putative alternative catalytic bases (S232 and Y536), the previously reported DTC-only variant (*Ag*AS:D621A) was utilized [16]. These residues were then mutated to simply remove the relevant hydroxyl—i.e., S232A and Y536F, either alone or in the context of Y287F or H348D. Any effect on product outcome was analyzed using a bacterial metabolic engineering system—i.e., expression in *Escherichia coli*, also engineered to produce **1** [21]. The DTC products are then observed via GC-MS analysis of hexane extracts from the resulting cultures as the primary alcohol derivatives (indicated by prime notation—e.g., copalol, **2′**), due to dephosphorylation by endogenous phosphatases, and identified by comparison with authentic standards. As expected, the S232A and Y536F mutations do not significantly shift product outcome on their own—i.e., these both predominantly yield **2** (Supplementary Figure S4). However, these substitutions had distinct effects on the LPP (**3**) producing variants.

**Figure 4:**
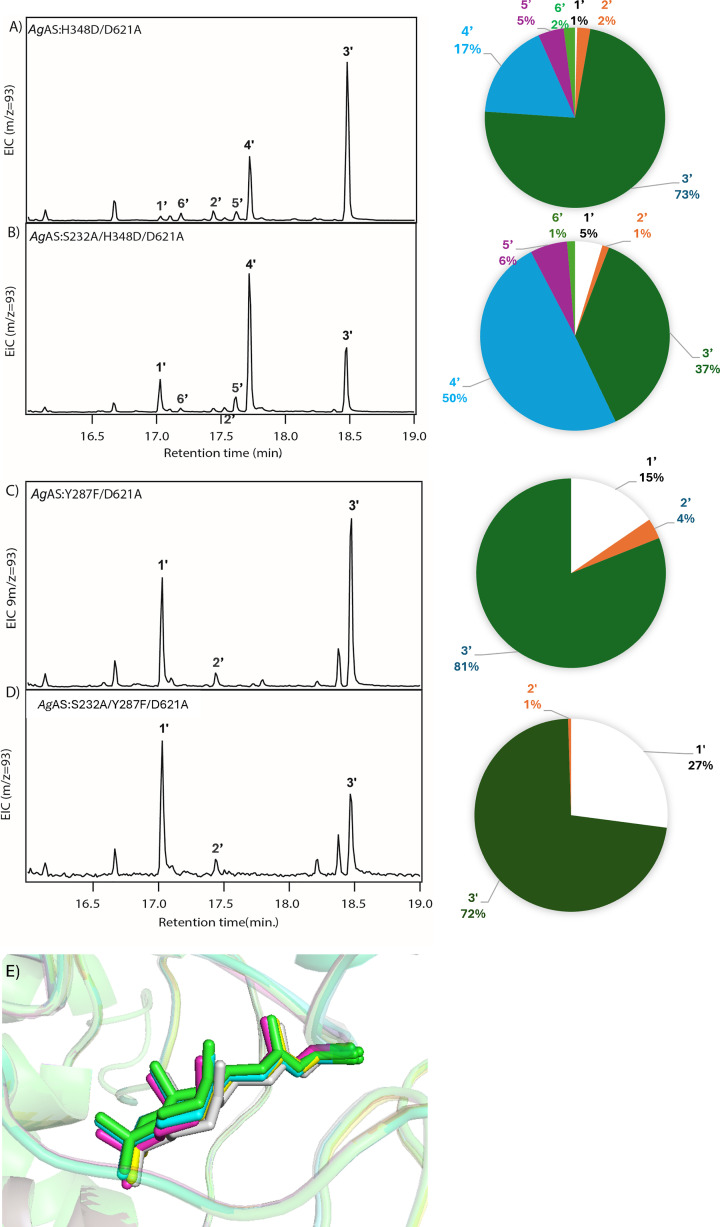
Investigating the role of S232 in the production of LPP. Enzymatic activity and structural analysis of *Ag*AS variants examining the role of S232 in the production of LPP (**3**). (**A–D**) GC-MS analysis, with shown extracted ion count (EIC) chromatograms and derived pie charts indicating relative product outcomes observed from total ion count peak areas from the indicated variant. Relative to (**A**) H348D or (**C**) Y287F, the addition of S232A ((**B**) and (**D**), respectively) validates serine as an alternative catalytic base via reduced production of **3**. (**E**) Representative overlayed poses of Y287F (yellow), H348D (green), S232A/Y287F (red), and S232A/H348D (blue) variants relative to the usual use of Y287 as the catalytic base (gray), which indicates little variation in the orientation of intermediate **A** in the active site upon water binding. A single angle constraint (allowed between 80 to 120 degrees) was added between **A** and Y287 or the mutant F287 to prevent **A** from rotating freely during the simulation.

In the case of S232A, when combined with H348D, a change in product outcome was observed, with significantly less **3** but greater amounts of **4** as well as a smaller increase in the double-bond isomer 8-endo-CPP (**5**), which is a trace product in both variants along with the rearranged halima-5,13*E*-dien-15-PP (**6**) (Figure 4). By contrast, S232A had less effect when combined with Y287F, as there is no significant shift in product ratio (Figure 4). However, this also led to somewhat reduced activity as evidenced by the increased accumulation of the dephosphorylated derivative of the substrate—i.e., (*E,E,E*)-geranylgeraniol (**1′**). Although visual inspection of the filtered poses revealed some differences in the positioning of **A** within the Y287F, H348D, S232A/Y287F, and S232A/H348D variants (constrained for the addition of water) compared with the usual reaction where Y287 serves as the catalytic base for the formation of **2** (Supplementary Figure S5), the introduction of a single angle constraint between **A** and Y287 (or F287) resulted in very close reactant alignment (Figure 4E). Such minor differences in orientation of **A** are consistent with the expectation that these variants, while necessarily accommodating a reactive water molecule, only lead to subtle effects on substrate positioning and overall reactant geometry relative to wild-type. Notably, such variance in positioning is consistent with the observed range of product outcomes.

While the experimental results with the S232A/H348D variant are consistent with the *TerDockin* prediction in partial reduction of the production of **3**, those with the S232A/Y287F variant do not seem to be. However, it should be noted that bicyclization of **1** (e.g., to **A**) is strongly energetically favorable [22], and the reactivity of carbocations leads to ready alkylation in the absence of a functional group suitably positioned for deprotonation [23], which may underlie the observed reduced activity with *Ag*AS:S232A/Y287F. Regardless, the results support *TerDockin*-based identification of S232 as a potential catalytic base contributing to the production of **3** in at least some cases.

**Figure 5:**
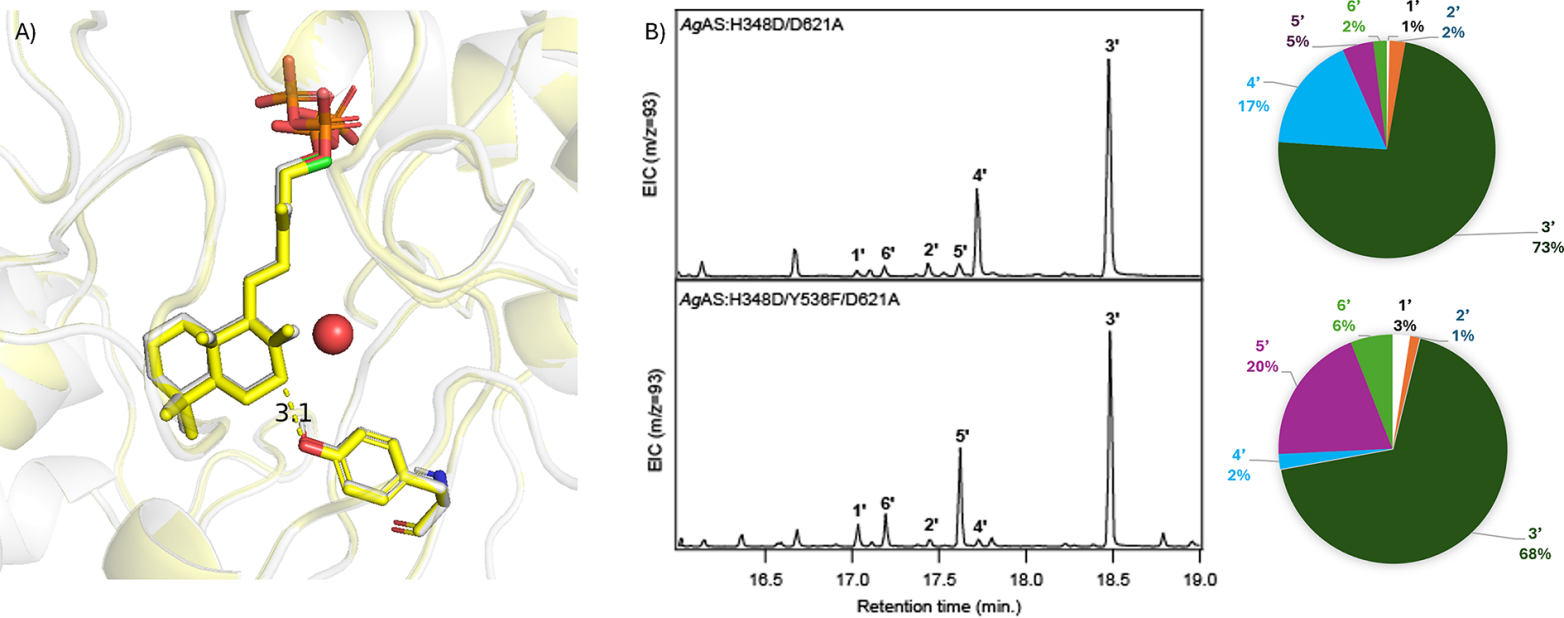
Y536 as the catalytic base for production of 7-endo-CPP. Role of Y536 in production of 7-endo-CPP (**4**) by *Ag*AS:H348D variant. (**A**) Y536 is positioned as a candidate base for the production of **4** in *Ag*AS:H348D/D621A (yellow), including the water (red sphere) otherwise positioned for addition, overlayed with wild-type *Ag*AS where Y287 acts as the base (grey). (**B**) GC-MS analysis, with shown EIC chromatograms and pie charts indicating relative product outcomes observed from total ion count peak areas from the indicated variants.

In H348D, Y536 appears to be a potential candidate for the catalytic base producing **4** in the *Ag*AS:H348D variant (Figure 5A). Indeed, the *Ag*AS:H348D/Y536F variant no longer produced significant amounts of **4**. Instead, while **3** continued to be the predominant product, this is now accompanied by substantial levels of **5**, as well as small amounts of **6**, and only trace amounts of **2** and **4** (Figure 5B). These results validate identification of Y536 as the primary catalytic base for deprotonation of **A** at C7 (i.e., for production of **4**) in the H348D variant by *TerDockin*. In addition, the predominant production of **3** by *Ag*AS:H348D/Y536F also is consistent with the *TerDockin* analysis, in that it excluded Y536 as a potential base for deprotonation of **B** to produce **3**.

**Figure 6:**
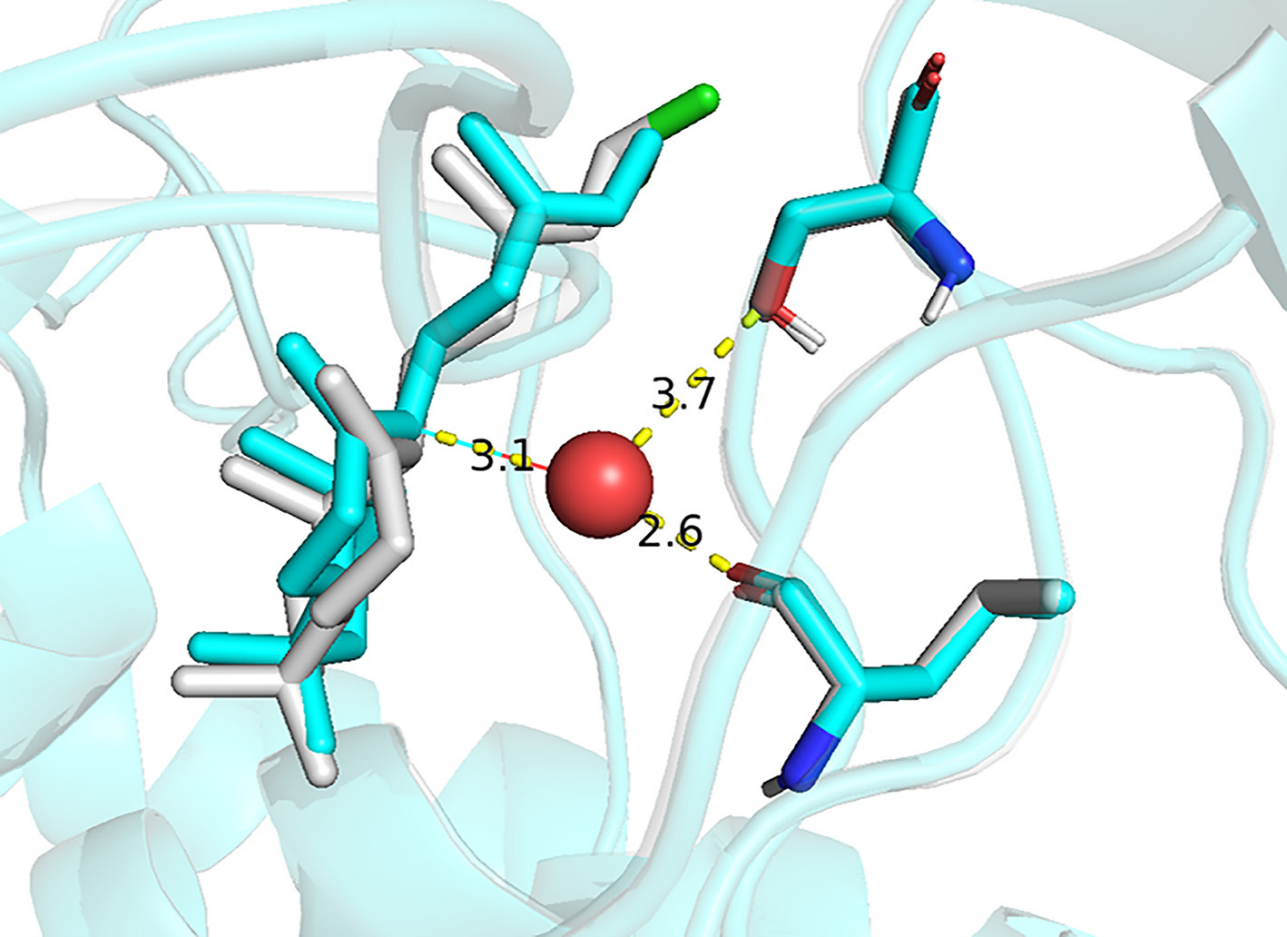
Water as the catalytic base for production of 8-endo-CPP. Water (red sphere) in *Ag*AS:H348D/Y536F/D621A may act as the general base for production of 8-endo-CPP (**5**), with potential activation by hydrogen bonds from S232 and L531 (blue), overlayed with the usual production of CPP (**2**) with Y287 acting as the base (grey).

To identify the catalytic base responsible for the production of **5** in the H348D variant, the energetically favorable poses for such deprotonation of **A** were closely examined, but no functional group within 5 Å of C9 was found. Consequently, the included water molecule, which might serve such a role, particularly if activated by hydrogen-bond partners, as has been observed in other DTCs [24], was considered. Indeed, upon constraining the water molecule to serve as the catalytic base for production of **5**, it was found to form hydrogen bonds with the backbone carbonyl of L531 and the side-chain hydroxyl group of S232 (Figure 6; see Supplementary Table S6 for constraints, which included an additional angular constraint between the ligand and Y287 to prevent rotation of **A**).

## Conclusions

Here, the *TerDockin* approach was utilized to examine the DTC activity of *Ag*AS as a model for the TPS-d3 subfamily involved in conifer resin acid biosynthesis. This enabled resolution of the catalytic base dyad, revealing that Y287, with activation from H348, serves to deprotonate the carbocation intermediate **A** at C17 to generate CPP (**2**). Further application to the known alteration of product outcome in the Y287F and H348D variants provides some insight into the observed production of the hydroxylated derivative LPP (**3**), establishing that S232 acts as a secondary base for production of **3**, at least to some extent, while Y536 acts as the primary base for the production of the olefinic isomer 7-endo-CPP (**4**) by *Ag*AS:H348D. Notably, while it has been observed that the catalytic base dyad residues are conserved within L**Y**S and PC**H** motifs in those TPS-d3 subfamily members producing **2** [6], building on recent investigation of DTC conservation [25], it is now evident that S232 is even more broadly conserved in this subfamily. Indeed, this includes a more recently discovered member from *Taiwania cryptomerioides* that naturally produces **3** [26] and which further contains an asparagine in place of the catalytic base dyad histidine (e.g., H348 in *Ag*AS), suggesting such substitution may be predictive of product outcome. While these results contrast with the invariant use of a tightly bound water as the catalytic base for production of *enantiomeric* products (i.e., *ent*-CPP and *ent*-LPP) by the DTCs required for gibberellin phytohormone biosynthesis and the relevant variants [27], it presumably reflects the ancient phylogenetic split between the TPS-d3 and TPS-c subfamilies [6]. Regardless, these results showcase the utility of *TerDockin* for investigating such terpene synthase/cyclase structure–function relationships, which can further guide work aiming to elucidate their evolution [28], and might enable rational redesign to access novel products [29].

## Methods

### General

All reagents were purchased from Fisher Scientific unless otherwise mentioned.

### Recombinant constructs and site-directed mutagenesis

All vectors used in the present study were constructed using the Invitrogen Gateway System. The parental DTC-only (*Ag*AS:D621A) construct has been previously described [21]. Mutagenesis was performed on pENTR/SD/D-TOPO constructs using whole-plasmid PCR with overlapping primers. The resulting variants were confirmed through whole-gene sequencing conducted by the DNA facility at Iowa State University. Verified mutants were subsequently transferred into pDEST14 using LR clonase reactions.

### Metabolic engineering

*Ex vivo* analysis of relevant expression constructs was carried out via metabolic engineering as previously described [6]. Briefly, each construct was individually introduced into the C41 OverExpress strain of *E. coli* (Lucigen) in combination with the pGG vector, which expresses a GGPP synthase. The resulting recombinant strains were grown in a pre-culture containing 5 ml of NZY media and the appropriate antibiotics in a plastic culture tube under 180 rpm and 37°C conditions for 16 h. The pre-culture was then transferred to 45 ml of TB media, containing 100 mM of phosphate buffer (pH 7.0) and antibiotics in 250 ml Erlenmeyer flasks, and grown (180 rpm and 37°C) until an OD_600_ of 0.6–0.8 was reached. The temperature was then lowered to 16°C for 1 h, and the cultures were then induced with 1 mM IPTG. The cultures were grown for an additional 72 h (180 rpm and 16°C). The enzymatic products were extracted by adding 50 ml hexanes and shaking for 20 min at 180 rpm and 37°C. After initial removal of the organic (hexanes) layer, 0.2 ml of ethanol was added to the remaining emulsion to facilitate further separation and repeated a second time if necessary (i.e., to obtain >49 ml of organic extract). The hexanes extract was divided equally between three glass test tubes, dried with a stream of concentrated N_2_ gas, resuspended in 600 μl of hexanes, and transferred to a vial for analysis via gas chromatography with mass spectral detection (GC-MS).

### GC-MS analysis

The GC-MS equipment and parameters used for all analyses were as previously described [30]. Briefly, the GC-MS equipment and parameters used for all analyses were as follows: an 8890 GC system equipped with a 5977B mass spectrometer (Agilent) operating in 70 eV electron ionization mode and using an HP-5MS column at a flow rate of 1.1 ml/min of helium. Samples were injected in spitless mode at a temperature of 250°C using a 7650A automatic liquid sampler. The oven temperature was maintained at 50°C for 3 min, followed by a ramp rate of 15°C/min to 300°C, which was maintained for an additional 3 min. Data from the mass spectrometer were recorded for mass-to-charge (m/z) ratios between 90 and 600, starting 13 min after sample injection until the end of the run.

### Quantum mechanical calculations

#### Energy-minimized structures

Electronic structure (QM) calculations were conducted using Gaussian 16 at the mPW1PW91/6-31+G(d,p) level of theory in the gas phase [31,32]. This approach has been previously validated for terpene-forming carbocation reactions [33–35] and terpene synthases more directly [36]. Intermediates underwent a conformational search using CREST at the GFN2-xTB level [37]. The resulting conformers were optimized at the mPW1PW91/6-31+G(d,p) level of theory. All conformers within 5 kcal/mol of the lowest energy structure were kept. To ensure accuracy and minimize computational cost, conformers were then filtered for an extended alkyl arm configuration, based on the crystal structure of the *At*CPS complexed with the substrate analog (S)-15-aza-14,15-dihydrogeranylgeranylthiolopyrophosphate (PDB ID: 4LIX) [38]. Resulting conformers were stored in a conformer library for docking.

The carbon skeleton was generated using the abovementioned QM approach to model the full ligand during docking, with a chlorine atom replacing the pyrophosphate group [39]. During docking, angle and distance constraints were applied to ensure proper attachment of the pyrophosphate and alignment of its oxygen with the chlorine atom (Supplementary Table S1).

#### Theozyme modeling

A theozyme approach was applied using the mPW1PW91/6-31+g(d,p) level of theory, a scan in which the N–H–C bond distances between imidazole and methylcyclohexane (mimicking deprotonation at carbon-17 (C17) of intermediate **A** by H348) were varied while the remainder of the structure was allowed to relax [40]. Multiple points along the potential energy ridge were optimized using a transition state optimization method [31,32], and the transition state was identified as a stationary point corresponding to a first-order saddle point by the presence of a single imaginary frequency. IRC calculations were performed to validate the connectivity of the predicted transition structure to the relevant reactant and product minima [41,42].

#### Docking calculations

The *TerDockin* approach [43,44], combined with the constrained FastRelax protocol in the Rosetta modeling suite, which allows reorientation of both reactant and nearby protein structure, was employed for docking into the crystal structure of *Ag*AS (RCSB PDB: 3S9V). Utilizing the ref2015 score function, a conformer library for the intermediate was docked into the protein through Rosetta, and geometry refinement was conducted using a Monte Carlo Metropolis sampling algorithm [45–48].

The known chemistry of class II DTC reactions was employed in the docking calculations by adding a set of constraints (Supplementary Tables S1 and S2). This included constraining D404 to C3 of **A**, reflecting the initiating protonation of the terminal C=C π bond by this residue [13]. Additionally, N451 was constrained to form a hydrogen-bond with D404, as this seems to be important for activating D404 to serve as the catalytic acid [49]. H348 or Y287 were constrained to C17 to simulate service as a catalytic base, where retained the H348 and Y287 residues were constrained to maintain the hydrogen bond observed in the crystal structure (Supplementary Tables S3 and S4). Water addition to form **3** was modeled by constraining a reactant water to C8 in **A** (Supplementary Table S5). Water was separately constrained for deprotonation of C9 in **A** (Supplementary Table S6).

Docking was carried out by the initial generation of 2500 poses, which were then subjected to a stepwise filtration process for each scenario. First, only those with constraint energies <1 (i.e., minimal deviation from any of the assigned constraints) were retained. Next, the dataset was further refined by selecting the lowest scoring 50% based on the total protein energy score. Finally, poses ranking within the lowest 10% for protein-reactant interface energy score were shortlisted. The remaining poses were then visually inspected using *Pymol* to assess their structural relevance (i.e., for the identification of putative catalytic bases), although this was not utilized as a filter.

## Supplementary Material

Supplementary Figures S1-S6 and Tables S1-S6

## Data Availability

The data from the *TerDockin* analyses has been deposited and is publicly accessible at https://doi.10.25380/iastate.31075468 [50].

## Open Access

Open access for this article was enabled by the participation of Iowa State University in an all-inclusive Read & Publish agreement with Portland Press and the Biochemical Society.

## Abbreviations

*Ag*AS: abietaenol synthase
*At*CPS: *Arabidopsis thaliana ent*-copalyl pyrophosphate synthase
CPP: copalyl pyrophosphate
DTCs: class II diterpene cyclases
DTS: class I diterpene synthase
EIC: extracted ion count
GC-MS: gas chromatography with mass spectral detection
GGPP: (E,E,E)-geranylgeranyl pyrophosphate
IRC: intrinsic reaction coordinate
LPP: labda-13-en-8α-ol-15-yl pyrophosphate
LRDs: labdane-related diterpenoids
m/z: mass-to-charge
TPS: terpene synthase
