# Supplementary material for: Peeking behind the carbocation: identification of (alternative) catalytic bases in the class II active site of conifer resin acid diterpene synthases
Source: Biochem J. 2026 Apr 28;483(5):819–29. doi: 10.1042/BCJ20250232 (PMC13142921; doi:10.1042/BCJ20250232)
Supplement: Supplementary Figures S1-S6 and Tables S1-S6 [file BCJ-2025-0232_supp.pdf]

<u>Supporting Information for</u>:

Peeking behind the carbocation: Identification of (alternative) catalytic bases in the class II active site of conifer resin acid diterpene synthases

Ahmed M.A.A. Raslan and Reuben J. Peters*

Roy J. Carver Department of Biochemistry, Biophysics & Molecular Biology, Iowa State University, Ames, IA 50011, USA

*Corresponding author: rjpeters@iastate.edu

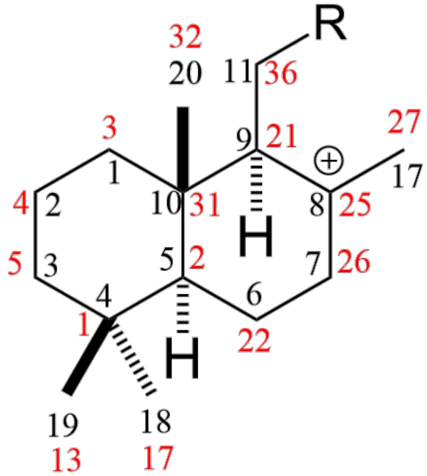

Figure S1: Gaussian numbering used in TerDockin simulations (red) versus conventional numbering used in manuscript (black).

Table S1: Modeling diphosphate-terpene bond with Rosetta constraints and PPi-protein constraints for full ligand docking:

Atoms constrained	Type	Constraint Value
Cl (Terpene)–O7(PPi)	Distance	0.0± 0.1 Å
C45(Terpene)–O7(PPi)	Distance	1.8 ± 0.1Å
C45-C44(Terpene)–O7(PPi)	Angle	110.6 ± 5 ˚
C45(Terpene)–O7-P2(PPi)	Angle	124.8± 10.0 ˚
O4(PPi) – OE1(E235)	Distance	5 ± 0.5Å

Table S2: General constraints used for all docking modeling D404 protonating the terminal π bond of GGPP to initiate the cyclization reaction, a protein-protein constraint where N342 hydrogen bonds to D404, activating it to react with the terpene precursor and a protein-protein constraint where H348 H-bond to Y287 (This constraint was eliminated when either residue was mutated):

Atoms constrained	Type	Constraint Value
OD2 (D404) – C5 (terpene)	Distance	2.5± 0.5 Å
OD2 (D404) – C5-C4 (terpene)	Angle	109.0 ± 10.0˚
ND2 (N342) – OD1 (D404)	Distance	2.7 ± 0.3 Å
ND2-CG (N342) – OD1(D404)	Angle	113.0± 10.0 ˚
ND2 (N342) – OD1-CG(D404)	Angle	131.2±10.0˚
ND1 (H348) – OH (Y287)	Distance	3.1± 0.3 Å
ND1-CE1(H348) – OH (Y287)	Angle	113.0±10.0˚
ND1 (H348) – OH-CZ (Y287)	Angle	123.2±10.0˚

Table S3: Y287 modeled to act as a base constraint:

Atoms constrained	Type	Constraint Value
C27(terpene) – OH(Y287)	Distance	3 ± 0.5 Å
C27-C25(terpene) – OH(Y287)	Angle	110.96 ± 10.0˚
C27(terpene)– OH-CZ (Y287)	Angle	115.89 ± 10.0˚

Table S4: H348 modeled to act as a base constraint:

Atoms constrained	Type	Constraint Value
C27(terpene) – ND1(H348)	Distance	3 ± 0.5 Å
C27-C25(terpene) – ND1(H348)	Angle	73.96 ± 10.0˚
C27(terpene)– ND1-CG (H348)	Angle	122.52 ± 10.0˚

Table S5: Water addition constraint:

Atoms constrained	Type	Constraint Value
O (H2O) – C25(terpene)	Distance	3± 0.3Å
C25-C21(terpene) – O (H2O)	Angle	90.00 ± 10.0˚
C25-C21-C31(terpene)–O (H2O)	Dihedral	160 ± 10.0˚

Table S6: Water constraints for C9 deprotonation

Atoms constrained	Type	Constraint Value
O (H2O) – C21(terpene)	Distance	2.8± 0.3Å
C21-C25(terpene) – O (H2O)	Angle	110.00 ± 11.0˚

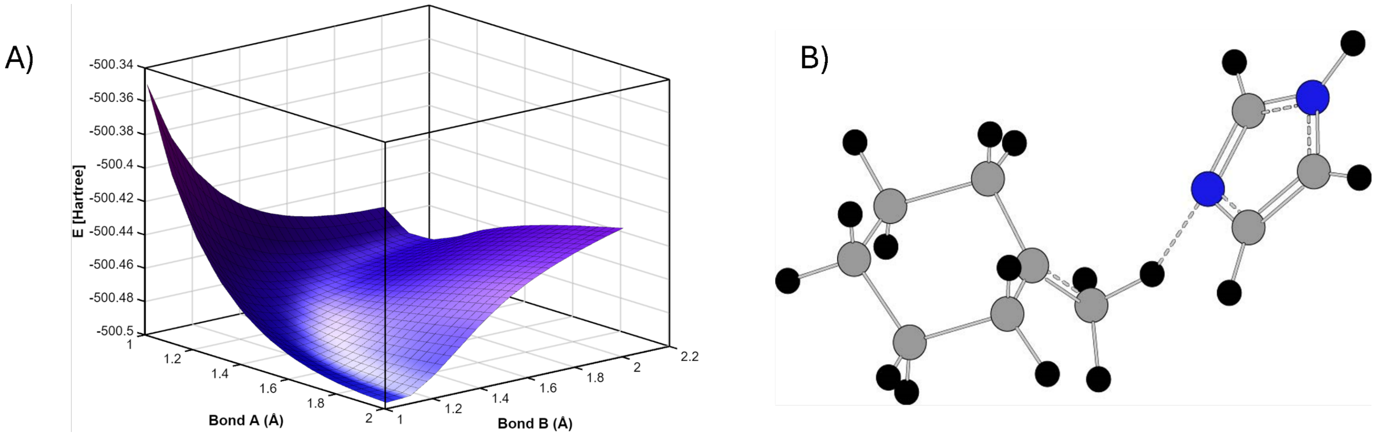

*Figure S2: A) 3D scan of N–H (bond A) and H–C (bond B) distances between imidazole and methylcyclohexane, modeling C17 deprotonation of intermediate **A** by H348. B) Model theozyme system to identify optimal angles for deprotonation by H348: imidazole and methylcyclohexane.*

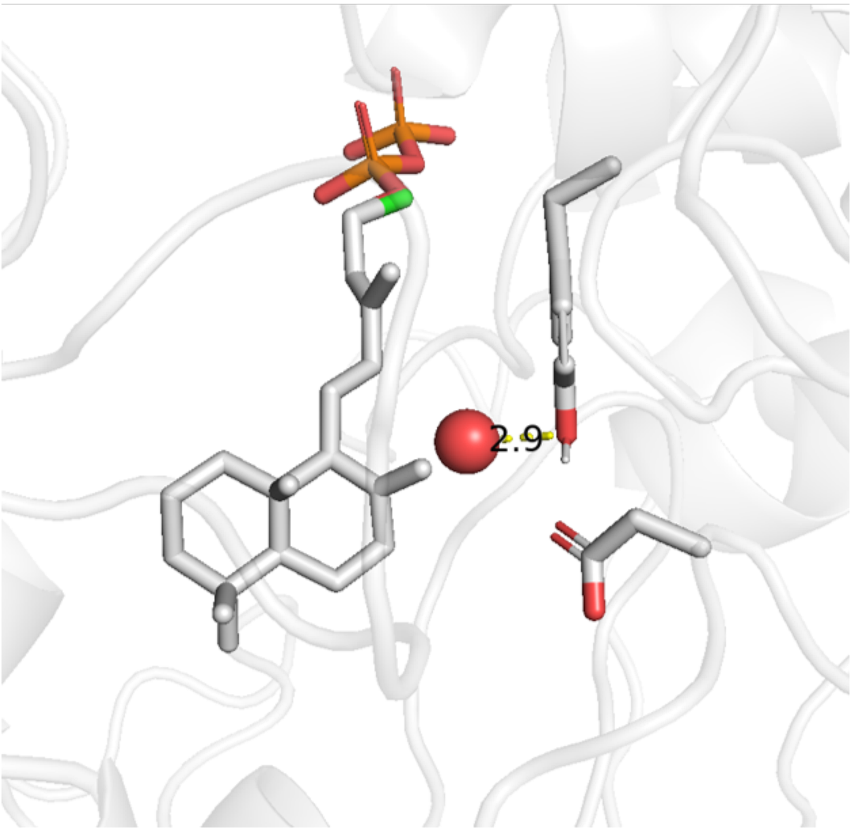

*Figure S3: In the H348D variant, production of **2** is blocked by the reactant water required for production of LPP (**3**), which forms a hydrogen bond with Y287 and prevents access to C17.*

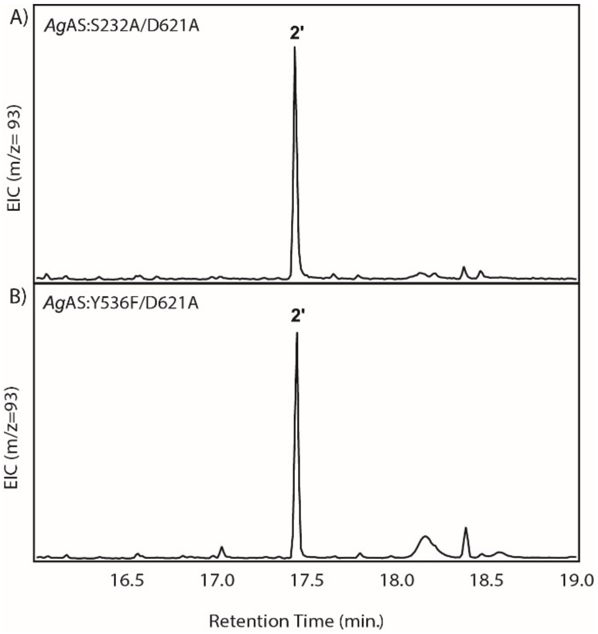

*Figure S4: Continued production of **2** from AgAS: S232A (A) or Y536F(B)*

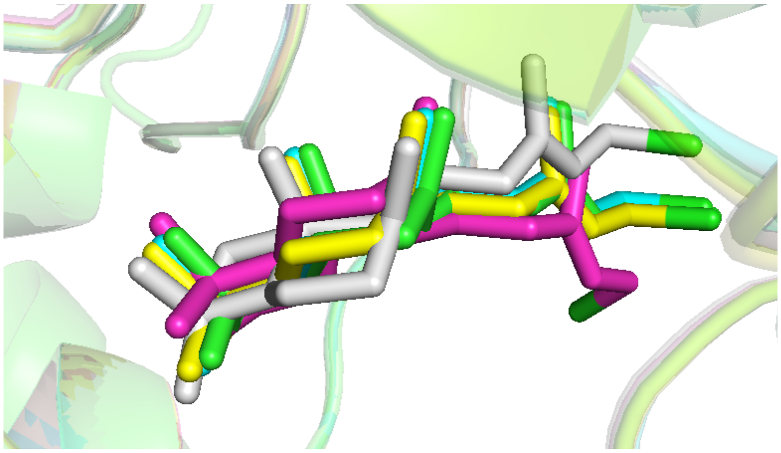

*Figure S5: Ligand orientation upon water addition relative to the wildtype enzyme with Y287 acting as the base for production of **2**.*
